# Type II Diabetes Mellitus and COVID-19: Exploring Insulin Management in Patients from Family Medicine Clinics

**DOI:** 10.3390/pharmacy13040093

**Published:** 2025-07-04

**Authors:** Chinemerem Opara, Annesha White, Kimberly G. Fulda, Somer Blair, Clare Aduwari, Nihitha Nukala, Yan Xiao

**Affiliations:** 1 College of Pharmacy, University of North Texas Health Science Center, 3500 Camp Bowie Blvd, Fort Worth, TX 76107, USA; annesha.white@unthsc.edu (A.W.); clareaduwari@yahoo.com (C.A.); nihithanukala@my.unthsc.edu (N.N.); 2 Texas College of Osteopathic Medicine, University of North Texas Health Science Center, 3500 Camp Bowie Blvd, Fort Worth, TX 76107, USA; kimberly.fulda@unthsc.edu; 3 JPS Health Network, 1500 S Main St, Fort Worth, TX 76104, USA; somerblair@yahoo.com; 4 College of Nursing and Health Innovation, University of Texas at Arlington, 701 S Nedderman Drive, Arlington, TX 76019, USA; yan.xiao@uta.edu

**Keywords:** diabetes, COVID-19, pandemic, insulin management

## Abstract

The COVID-19 pandemic disrupted routine care for individuals with type 2 diabetes mellitus (T2DM), raising concerns about its impact on glycemic control and medication management. This study evaluated the relationship between insulin use and glycemic control among T2DM patients during the pandemic. A retrospective analysis was conducted using deidentified clinical and prescription data from two family medicine clinics, comparing data from the pre-COVID-19 period (1 March 2019–13 March 2020) and during the COVID-19 pandemic (14 March 2020–31 March 2021). Patients included had at least two A1c values before the COVID and one during the COVID. A1c control was defined as less than 8%. Among 992 patients, 238 experienced a change in A1c status: 128 improved and 110 worsened. Mean A1c remained stable at 8.2 across both periods. A majority of patients who improved were using insulin during the COVID-19 era, although some discontinued insulin at some point during the study period. These findings suggest that consistent insulin therapy may have helped maintain glycemic control despite healthcare disruptions. This study highlights the importance of sustained medication management and suggests that integrating telehealth and pharmacist-led care could support diabetes control during future healthcare system challenges.

## 1. Introduction

Type 2 diabetes mellitus (T2DM) represents a significant public health challenge globally, characterized by insulin resistance and elevated blood glucose levels. Its prevalence continues to escalate, driven by factors like sedentary lifestyles, unhealthy dietary habits, and aging populations [1]. With this rise in prevalence comes a substantial economic burden associated with managing the condition, including expenses related to medications, hospitalizations, and complications [2]. The estimated cost of diabetes in the United States exceeds $400 billion [3]. To address the complexities of T2DM, healthcare organizations and professional associations like the American Diabetes Association advocate for comprehensive management strategies [4]. These encompass regular glucose monitoring, adherence to healthy dietary patterns, engagement in physical activity, and consistent medical follow-ups. Such interventions aim not only to control blood glucose levels but also to mitigate the risk of debilitating complications such as cardiovascular disease, neuropathy, and retinopathy [5]. Additional interventions to reduce complications may be needed due to the COVID-19 pandemic.

Emerging evidence has revealed that COVID-19 not only exacerbates existing diabetes but may also contribute to the onset of new cases [1,4]. SARS-CoV-2, the virus responsible for COVID-19, can impair pancreatic function by binding to angiotensin-converting enzyme 2 (ACE2) receptors expressed in pancreatic islet cells, potentially leading to β-cell dysfunction and hyperglycemia. Additionally, the systemic inflammatory response associated with COVID-19 can induce insulin resistance, further complicating glycemic control in individuals with or without pre-existing diabetes. These mechanisms underscore the bidirectional relationship between COVID-19 and diabetes, highlighting the need for vigilant glycemic monitoring during and after infection.

Beyond these biological mechanisms, the pandemic also introduced significant systemic barriers to diabetes management. The onset of the COVID-19 pandemic in March 2020 triggered widespread disruptions across healthcare systems worldwide, posing challenges for individuals managing T2DM. The implementation of public health measures, including lockdowns and social distancing protocols, potentially hindered access to routine medical care and medications, exacerbating the difficulties faced by diabetes patients [4]. Moreover, individuals with T2DM are at increased risk for severe COVID-19 outcomes, including hospitalization and mortality [5]. The interplay between diabetes and COVID-19 exacerbates the urgency of effectively managing blood glucose levels and adopting stringent infection prevention measures. Specifically, for Medicare patients, there is a tremendous economic and clinical impact. Approximately 25% of Americans aged 65 and older have type 2 diabetes, costing $205 billion for medical care [6]. A study conducted on a Medicare population of 173,942 diabetic patients with COVID-19 in 2020, compared younger adults with diabetes to older adults who reported having less access to care [7].

Disruptions in daily routines, limited access to diabetic medications such as insulin, and psychological distress contribute to suboptimal self-care behaviors and worsened glycemic control. On the other hand, the expansion of telehealth and self-care management strategies during the pandemic helped some patients improve glycemic control [5].

While several studies have examined the impact of the COVID-19 pandemic on diabetes care broadly, few have focused specifically on insulin management within safety-net primary care settings. These clinics serve vulnerable populations who may face greater barriers to consistent care, medication access, and telehealth utilization. Our study addresses this gap by exploring how patterns of insulin use, particularly continuity or discontinuation, were associated with glycemic control among patients with T2DM during the pandemic. By focusing on real-world data from safety-net family medicine clinics, this research provides novel insights into diabetes management under conditions of healthcare disruption.

The objective of this retrospective cohort study is to assess the association between insulin use and glycemic control, as measured by changes in hemoglobin A1c (A1c) levels, among patients with type 2 diabetes mellitus during the COVID-19 pandemic. This study also explores patterns of medication use that may have contributed to changes in A1c control.

## 2. Materials and Methods

### 2.1. Study Design

This retrospective observational study utilized de-identified electronic health record data from two family medicine clinics. The study was conducted in accordance with the Declaration of Helsinki and was approved by the Ethics Committee of the University of Texas at Arlington and the North Texas Regional Institutional Review Boards (Protocol #2019-0439, approved 11 October 2019).

Although the overall design was a retrospective cohort study, we conducted a descriptive subgroup analysis on 238 patients whose A1c control status changed between the pre-COVID-19 (1 March 2019–13 March 2020) and COVID-19 (14 March 2020–31 March 2021) periods. This analysis was previously referred to as a “case series review,” but is now more accurately described as a descriptive analysis within the cohort.

Data were extracted for patients aged 18 years or older as of March 1, 2019, and included demographics (sex, race, ethnicity, insurance status, and preferred language), hemoglobin A1c values with corresponding dates, visit dates (including telehealth), and medication prescriptions. Telehealth visits were identified using visit type codes in the electronic health record. However, data on provider type (e.g., physician, nurse practitioner, and pharmacist) and visit content were not available and were therefore not analyzed in relation to glycemic outcomes.

Patients were eligible for inclusion if they had a diagnosis of type 2 diabetes and at least two A1c values in the pre-COVID-19 period and one during the COVID-19 period. To assess care changes during the pandemic, we analyzed patients whose A1c status either improved (≥8% to <8%) or worsened (<8% to ≥8%) based on the last A1c value in each period.

Medication use patterns were categorized as: (1) insulin and other T2DM medications, (2) non-insulin medications only, or (3) insulin-only. A medication was considered “discontinued” if there was no subsequent prescription for the same drug or drug class, indicating a gap in therapy. Selected patient cases were included to illustrate changes in diabetes medication management during the COVID-19 era.

While the dataset provided detailed clinical and prescription information, it did not include patient-level data on potential confounders such as diet, physical activity, stress, or socio-economic status. These limitations are acknowledged in the interpretation of glycemic outcomes.

### 2.2. Study Outcomes

The primary outcome was change in glycemic control, defined by hemoglobin A1c (HbA1c) levels. Patients were categorized into four groups based on A1c status: (1) stayed controlled (<8% in both periods), (2) stayed uncontrolled (≥8% in both periods), (3) improved (≥8% to <8%), and (4) worsened (<8% to ≥8%). Secondary outcomes included patterns of insulin and non-insulin diabetes medication use during the COVID-19 period, with a focus on medication continuity and discontinuation.

### 2.3. Definitions

- A1c Control: Defined as HbA1c <8%, in accordance with the American College of Physicians’ guidance for outpatient diabetes management [8].
- Improved Glycemic Control: A1c decreased from ≥8% in the pre-COVID period to <8% during the COVID-19 period.
- Worsened Glycemic Control: A1c increased from <8% in the pre-COVID period to ≥8% during the COVID-19 period.
- Medication Discontinuation: Defined as the absence of a renewed prescription for a drug or drug class during the COVID-19 period. Discontinuation was inferred from a gap in prescriptions without a subsequent refill or renewal. Refill data were not available; therefore, actual adherence could not be directly assessed.
- Insulin Use Categories: Patients were grouped into three categories based on their medication regimen during the COVID-19 period: (1) insulin only, (2) insulin plus other diabetes medications, and (3) non-insulin medications only.

### 2.4. Statistical Analysis

Descriptive statistics were calculated to evaluate differences in baseline characteristics between cohorts with A1c control status change. For categorical variables (e.g., sex, race/ethnicity, and insurance status), Pearson’s Chi-Squared test was used to assess group differences. For continuous variables (e.g., age and A1c values), independent samples *t*-tests were applied. Statistical significance was defined as a two-tailed *p*-value of <0.05. For calculations involving A1c values, the last A1c value in the pre-COVID-19 era and the last A1c value in the COVID-19 era were used per patient. All statistical analyses were computed using R version 4.2.2. 

## 3. Results

### 3.1. Baseline Characteristics

Among 992 patients who visited the study health center and were included as study participants, a total of 128 individuals got better (i.e., A1c decreased from ≥8% to <8%), from pre to during COVID-19 and 110 got worse (i.e., A1c increased from <8% to ≥8% or higher). Out of the total cohort, there were slightly more females (54.5%). The majority specified as Hispanic (36.5%) or non-Hispanic Black (33.2%) in terms of race/ethnicity and spoke English versus non-English (64.7% vs. 35.3%) as their primary language. Notably, there were slight differences in insurance status at baseline between those who improved and those who worsened; for example, 5.5% of patients who worsened had self-pay versus 10.9% of those who improved. There were no statistically significant differences between the two groups (Table 1). From the pre-COVID-19 to the COVID-19 era, there was a 2% decrease in the number of patients with A1c greater than 8%. There was no change in the mean A1c from the pre-COVID-19 cohort compared to the COVID-19 era cohort, as seen in Table 2 below.

To illustrate these patterns, Table 3 presents six randomly selected patient cases showing changes in A1c and medication use. Most patients used insulin throughout the COVID-19 period and experienced adjustments to their diabetes medication regimens. These changes included both the initiation and titration of medications, with many prescriptions beginning during the COVID-19 era.

A descriptive subgroup analysis was conducted on 238 patients whose A1c control status changed, either improved or worsened. Among the subset of 110 individuals whose A1c control worsened, 60 (54.7%) patients remained on insulin therapy consistently throughout the study period. Within this cohort, 23 (21%) discontinued insulin entirely, while 38 (35%) discontinued at least one non-insulin diabetes medication. In contrast, among the 128 patients whose A1c control improved, 90 (71%) consistently used insulin throughout the study period, while 37 (29%) patients did not use insulin during the study period. Notably, 46 out of these 128 (36%) patients discontinued insulin at some point, and 66 (52%) discontinued at least one non-insulin medication.

To illustrate these patterns, Table 3 presents six randomly selected patient cases showing changes in A1c and medication use. Most patients used insulin throughout the COVID-19 period and experienced adjustments to their diabetes medication regimens. These changes included both initiation and titration of medications, with many prescriptions beginning during the COVID-19 era (See Supplementary Materials for a full list of all patient cases reviewed).

### 3.2. Glycemic Control Outcomes

Of the 992 patients included in the study, 128 (12.9%) experienced improved glycemic control, defined as a decrease in A1c from ≥8% pre-COVID to <8% during the COVID-19 period. Conversely, 110 patients (11.1%) experienced worsened control, with A1c increasing from <8% to ≥8%. The remaining patients either maintained controlled (<8%) or uncontrolled (≥8%) A1c levels across both time periods.

The mean A1c remained stable at 8.2% in both the pre-COVID-19 and COVID-19 periods, while the median A1c was consistent at 7.6% (Table 2). Overall, there was a modest 2% reduction in the proportion of patients with A1c values above 8% from the pre-COVID-19 to the COVID-19 era.

### 3.3. Medication Use Patterns

A descriptive review was conducted on the 238 patients whose A1c status changed between the pre-COVID-19 and COVID-19 periods. Among the 110 patients whose A1c worsened, 60 (54.7%) remained on insulin therapy throughout the study period. However, 23 (21%) discontinued insulin entirely, and 38 (35%) discontinued at least one non-insulin diabetes medication.

In contrast, of the 128 patients whose A1c improved, 90 (71%) consistently used insulin, while 37 (29%) did not use insulin during the study period. Notably, 46 (36%) of the improved group discontinued insulin at some point, and 66 (52%) discontinued at least one non-insulin medication.

Table 3 presents six illustrative patient cases that highlight variations in insulin and non-insulin medication use, as well as their corresponding changes in A1c levels. These examples underscore the potential impact of consistent medication use on glycemic control during periods of healthcare disruption, such as the COVID-19 pandemic.

## 4. Discussion

As in other studies of diabetic patients during the pandemic (9), we did not find a worsening of A1c levels despite disruptions in care among the diabetic patients in two safety net family clinics. We assessed the association between insulin use and glycemic control among T2DM patients during the COVID-19 pandemic to identify potential contributing factors. Our case series review showed that most patients whose A1c improved were on insulin, compared to those whose A1c worsened. During the COVID-19 era, this population experienced significant disruptions in diabetes management due to social distancing policies and clinic closures, and other pandemic-related challenges.

These findings reflect the multifactorial nature of diabetes management during a global health crisis. While insulin use was more common among patients who improved their A1c, its presence among those who worsened suggests that medication alone is not a sufficient predictor of glycemic control. This aligns with prior research indicating that factors such as patient engagement, access to care, and behavioral adaptations (e.g., diet, exercise, and stress management) significantly influence diabetes outcomes during periods of disruption [9,10].

The observed stability in mean A1c across the cohort may reflect selection bias toward patients who remained engaged with the healthcare system and had complete A1c data. This could mask more severe glycemic deterioration in patients who were lost to follow-up or lacked access to care. The subgroup analysis, therefore, provides a more nuanced view of how medication patterns, particularly discontinuation, correlate with changes in glycemic control.

These results underscore the importance of continuity in diabetes care, especially during emergencies. However, they also highlight the limitations of relying solely on prescription data to infer treatment effectiveness. Future studies should incorporate patient-reported outcomes, adherence measures, and social determinants of health to better contextualize clinical data. Prior studies have evaluated glycemic control in T2DM patients during the pandemic. One study [9] found that glycemic values in patients improved significantly during lockdowns, and researchers associated this with positive changes in self-care and digital diabetes management. Another study [10] found no clinically significant difference in medication adherence, blood glucose monitoring before and during the pandemic, but noted that clinic closures made glycemic control more difficult for many patients, despite the availability of telemedicine. Contributing factors included reduced physical activity and increased consumption of high-glycemic foods. Another factor that may have supported improved outcomes for some patients could be the increased utilization of telehealth [11]. The case examples in our study illustrated potential diabetes management differences among the patients with improved versus worsened A1c levels. Those with improved A1c levels maintained their treatment regimens consistently across the COVID-19 era. Conversely, among individuals in the worsened group, one patient stood out for discontinuing all medications following the onset of COVID-19, indicating a potential interruption in therapy.

In terms of treatment options, initiating insulin treatment becomes necessary when glycemic targets remain unattained despite 2–3 months of oral usage. Evidence suggests that the swift incorporation of insulin treatment leads to improved treatment outcomes and improved quality of life for affected individuals. As shown by our study, a significant portion of successfully treated patients were utilizing some form of insulin therapy, resulting in improved A1c. According to Swinnen, combining insulin with other oral agents can reduce injection frequency, simplify titration, and improve adherence. This integrated approach may enhance diabetes management while supporting patient comfort [12,13]. Our findings suggest that consistent insulin coverage may contribute to A1c control. Future research should explore the role of provider type in telehealth visits and assess outcomes, such as prescription pick-up, medication adherence, and patient follow-up care.

While our study found that patients who maintained consistent insulin therapy were more likely to experience improved A1c control, this association does not imply causation. A substantial proportion of patients whose A1c worsened were also on insulin, indicating that other unmeasured factors, such as medication adherence, insulin titration, lifestyle behaviors, and psychosocial stress, may have influenced outcomes. These findings highlight the need for a more comprehensive evaluation of diabetes management beyond medication use alone.

Alternative explanations for our findings include unmeasured variables such as changes in diet, physical activity, stress, or socio-economic status, which may have independently influenced glycemic outcomes. Additionally, some patients may have received care or prescriptions outside the study clinics, potentially affecting the accuracy of medication tracking.

### Limitations

This study has several limitations. First, the retrospective design limits the ability to draw causal inferences between insulin use and glycemic control. Second, comorbidity data were not extracted from the electronic health records and are, therefore, not included in the baseline characteristics. This restricts our ability to assess the influence of coexisting conditions on glycemic outcomes. Third, medication adherence was inferred from prescription records, which may not accurately reflect actual patient behavior. Prescription data indicate prescribing patterns but do not confirm whether medications were filled or taken as directed. Additionally, patients may have obtained medications from external providers or pharmacies not captured in the study’s electronic health record system, potentially leading to underestimation or misclassification of medication use.

Fourth, the reasons for medication changes, such as clinical decisions based on A1c trends or access-related issues, were not captured, making it difficult to determine whether changes were clinically driven or due to barriers in care. Fifth, the study may be subject to selection bias, as it included only patients with complete A1c data across both time periods, potentially excluding those with less consistent care or follow-up. Sixth, unmeasured confounding variables such as changes in diet, physical activity, stress levels, or socio-economic status during the pandemic may have influenced both insulin use and glycemic control but were not captured in the dataset.

Finally, within the subset of patients whose A1c levels surpassed the 8% threshold, there were instances where A1c demonstrated improvement but did not meet the predefined cutoff criteria for classification. These methodological constraints limit the strength of conclusions regarding the effectiveness of insulin therapy.

Despite these limitations, our findings underscore the critical role of consistent insulin therapy in maintaining glycemic control during healthcare disruptions. Future research should incorporate comorbidity profiles, patient-reported outcomes, and social determinants of health to provide a more comprehensive understanding of diabetes management in crisis settings. Additionally, evaluating the impact of provider type, telehealth engagement, and pharmacist-led care could inform more resilient and equitable care models.

## 5. Conclusions

The current study highlights prescription patterns for type 2 diabetes medications during the pre-COVID-19 and COVID-19 periods. The comprehensive review of patient cases revealed that among individuals with previously uncontrolled diabetes who achieved improved A1c levels, the majority were utilizing insulin consistently throughout the pandemic. These findings offer valuable insights into insulin management during a period of significant healthcare disruption. Despite widespread disruptions in healthcare access, the overall glycemic control remained stable across the study population, suggesting that continuity in insulin therapy may have played a protective role.

Importantly, patients who demonstrated improved glycemic outcomes were more likely to have sustained their insulin regimens, underscoring the critical role of insulin in diabetes management during crises. As healthcare systems transition into post-pandemic models of care, integrating clinical pharmacists into multidisciplinary teams and expanding telehealth services may enhance medication adherence, patient education, and overall diabetes management.

While the association between consistent insulin use and improved A1c is compelling, this study did not directly evaluate the impact of telemedicine, pharmacist-led interventions, or other support systems. Future research should investigate these elements, along with provider type, telehealth engagement, and social determinants of health, to inform more resilient and equitable care models.

## Figures and Tables

**Table 1:** Demographics of The Health Center Type 2 Diabetes Population.

Demographics	Overall	Stayed Controlled	Stayed Uncontrolled	Worsened	Improved	*p*-Value
		(N = 457)	(N = 297)	(N = 110)	(N = 128)	
Sex						0.35
Female	541 (54.5%)	252 (55.1%)	159 (53.5%)	56 (50.9%)	74 (57.8%)	
Male	451 (45.5%)	205 (44.9%)	138 (46.5%)	54 (49.1%)	54 (42.2%)	
Baseline Age	56.0 [49.5;63.0]	58.0 [51.0;64.0]	55.0 [47.0;61.0]	56.0 [50.0;63.0]	56.0 [48.5;63.0]	0.95
Race/Ethnicity						0.88
Hispanic	361 (36.5%)	155 (34.0%)	122 (41.4%)	38 (34.5%)	46 (35.9%)	
Non-Hispanic Black	328 (33.2%)	157 (34.4%)	92 (31.2%)	38 (34.5%)	41 (32.0%)	
Non-Hispanic Other	153 (15.5%)	83 (18.2%)	38 (12.9%)	16 (14.5%)	16 (12.5%)	
Non-Hispanic White	147 (14.9%)	61 (13.4%)	43 (14.6%)	18 (16.4%)	25 (19.5%)	
Primary Language						0.26
English	642 (64.7%)	293 (64.1%)	192 (64.6%)	68 (61.8%)	89 (69.5%)	
Not English	350 (35.3%)	164 (35.9%)	105 (35.4%)	42 (38.2%)	39 (30.5%)	
Insurance at Baseline						0.32
Charity	327 (33.0%)	128 (28.0%)	122 (41.1%)	41 (37.3%)	36 (28.1%)	
Commercial	156 (15.7%)	81 (17.7%)	34 (11.4%)	19 (17.3%)	22 (17.2%)	
Grants	6 (0.6%)	1 (0.2%)	5 (1.7%)	0 (0.0%)	0 (0.0%)	
Liability	1 (0.1%)	1 (0.2%)	0 (0.0%)	0 (0.0%)	0 (0.0%)	
Medicaid	149 (15.0%)	73 (16.0%)	51 (17.2%)	13 (11.8%)	12 (9.4%)	
Medicare	283 (28.5%)	147 (32.2%)	61 (20.5%)	31 (28.2%)	44 (34.4%)	
Self Pay	70 (7.1%)	26 (5.7%)	24 (8.1%)	6 (5.5%)	14 (10.9%)	

**Table 2:** Description of Last and Median A1c Pre-COVID-19 vs. COVID-19 Era (N = 992).

	Last A1c Greater than 8%	Median A1c (IQR)	Mean (SD)
Pre-COVID-19 A1c	425 (43%)	7.6 (2.5)	8.2 (2.1)
COVID-19 A1c	407 (41%)	7.6 (2.8)	8.2 (2.1)

**Table 3:** Case Examples for Patients with A1c Changes to Controlled or Uncontrolled Status.

Cases	Insulin Use Patterns	Prescription Pattern Description	A1c Change Pre- to COVID-19 Era
Uncontrolled—Patient Case 1	Insulin use only.	Insulin Long-acting insulin during the COVID-19 era. Other DM medications None.	A1c = 8.4–9.1
Uncontrolled—Patient Case 2	Insulin use and other DM medication use.	Insulin Kept one long-acting insulin during the COVID-19 era. Other DM medications Discontinued all prescriptions during the COVID-19 era that started in the pre-COVID-19 era.	A1c = 7.2–14.0
Uncontrolled—Patient Case 3	No insulin use, but other DM medication use.	Insulin None. Other DM medications Kept all prescriptions through the COVID-19 era.	A1c = 6.8–9.5
Controlled—Patient Case 1	Insulin use only.	Insulin Kept short-acting insulin during the COVID-19 era Other DM medications None.	A1c = 9.3–7.6
Controlled—Patient Case 2	Insulin use and other DM medications use	Insulin Kept short-acting and long-acting insulin prescriptions through the COVID-19 era. Other DM medications Kept one prescription from the pre-COVID-19 era through the COVID-19 era.	A1c = 11.8–7.8
Controlled -Patient Case 3	No insulin use, but other DM medication use	Insulin None. Other DM medications Kept one prescription from the pre-COVID-19 era through the COVID-19 era.	A1c = 11.1–7.4

## Data Availability

The original contributions presented in this study are included in the article/Supplementary Materials. Further inquiries can be directed to the corresponding author.

## Supplementary Materials

The following supporting information can be downloaded at: https://www.mdpi.com/article/10.3390/pharmacy13040093/s1, Table S1: Case Examples for Patients with A1c Changes to Controlled or Uncontrolled Status, and Table S2: Case Examples for Patients with A1c Changes to Uncontrolled Status.
