# Supplementary material for: Type II Diabetes Mellitus and COVID-19: Exploring Insulin Management in Patients from Family Medicine Clinics
Source: Pharmacy (Basel). 2025 Jul 4;13(4):93. doi: 10.3390/pharmacy13040093 (PMC12286016; doi:10.3390/pharmacy13040093)
Supplement: Supplementary file 1 [file pharmacy-13-00093-s001.zip › pharmacy-3699048-supplementary.pdf]

Table S1: Case Examples for Patients with A1c Changes to Controlled or Uncontrolled Status

Case ID	Insulin Use Pattern	Prescription Pattern Descriptions	Number of Prescriptions Discontinued	A1C Change Pre COVID to COVID era
Case #1	No Insulin use, but other DM medications used	Insulin<br>None<br><br>Other DM medications<br>Glipizide and Metformin	0	8.1-7.6
Case #2	No Insulin use, but other DM medications used	Insulin<br>None<br><br>Other DM medications<br>glipizide, pioglitazone and metformin	1	10.7-7.7
Case #3	No Insulin use, but other DM medications used	Insulin<br>None<br><br>Other DM medications<br>glipizide and metformin	0	9.9-7.4
Case #4	No Insulin use, but other DM medications used	Insulin<br>None<br>Other DM medications<br>glipizide and metformin	0	7.0-7.5
Case #5	Insulin use and other DM medication used	Insulin<br>Aspart and Levemir<br><br>Other DM medications<br>Trulicity, Januvia and metformin	1	7.8-6.6
Case #6	No Insulin use, but other DM medications used	Insulin<br>None<br><br>Other DM medications<br>glimepiride	0	7.1-6.8
Case #7	Insulin use and other DM medication used	Insulin<br>Novolog<br><br>Other DM medications<br>Trulicity, pioglitazone, and glipizide	2	8.6-7.0
Case #8	Insulin use and other DM medication used	Insulin<br>Levemir<br><br>Other DM medications<br>metformin	2	7.4-7.5

Case #9	Insulin use only	Insulin<br>glargine and Lispro<br>Other DM medications<br>None	0	7.7-7.0
Case #10	Insulin use and other DM medication used:	Insulin<br>Levemir, Aspart, and Lispro<br><br>Other DM medications<br>pioglitazone and Victoza	1	8.5-6.8
Case #11	No Insulin use, but other DM medications used	Insulin<br>None<br>Other DM medications<br>metformin	1	8.0-7.1
Case #12	No Insulin use, but other DM medications used	Insulin<br>None<br>Other DM medications<br>metformin and glipizide	0	9.4-7.7
Case #13	Insulin use and other DM medication used	Insulin<br>Levemir, Aspart, and Novolin, and Lantus<br><br>Other DM medications<br>metformin	1	9.8-7.3
Case #14	Insulin use and other DM medication used	Insulin<br>Lantus, Lispro and Humalog<br><br>Other DM medications<br>metformin	2	8.8-8.0
Case #15	Insulin use and other DM medication used	Insulin<br>Levemir, Lispro and Humalog<br><br>Other DM medications<br>Trulicity	1	11.6-7.6
Case #16	Insulin use and other DM medication used	Insulin<br>Levemir<br>Other DM medications<br>metformin	0	8.0-8.0
Case #17	No Insulin use, but other DM medications used	Insulin<br>Other DM medications<br>metformin and glipizide	0	8.9-7.4
Case #18	Insulin use and other DM medication used	Insulin<br>Aspart and Levemir<br>Other DM medications<br>metformin and glipizide	2	9.6-7.3
Case #19	Insulin use only	Insulin<br>glargine and Aspart<br>Other DM medications<br>None	0	8.0-8.0

Case # 20	Insulin use and other DM medication used	<u>Insulin</u><br>Levemir<br><u>Other DM medications</u><br>metformin and glimepiride	0	11.3-5.7
Case #21	No Insulin use, but other DM medications used	<u>Insulin</u><br>None<br><u>Other DM medications</u><br>metformin and glipizide	1	9.1-7.6
Case #22	No Insulin use, but other DM medications used	<u>Insulin</u><br>None<br><u>Other DM medications</u><br>metformin, Onglyza and glipizide	2	8.5-6.4
Case #23	No Insulin use, but other DM medications used	<u>Insulin</u><br>None<br><u>Other DM medications</u><br>metformin, Jardiance, pioglitazone, glipizide, and Januvia	0	7.5-6.5
Case #24	No Insulin use, but other DM medications used	<u>Insulin</u><br>None<br><u>Other DM medications</u><br>Tradjenta, Jentadueto, and Jardiance	2	10.0-6.6
Case #25	Insulin use and other DM medication used	<u>Insulin</u><br>Levemir and Lispro<br><u>Other DM medications</u><br>metformin	1	9.0-7.7
Case #26	Insulin use only	<u>Insulin</u><br>Aspart, glargine and Lispro<br><u>Other DM medications</u><br>None	1	8.6-7.2
Case #27	Insulin use and other DM medication used	<u>Insulin</u><br>Levemir and Aspart<br><u>Other DM medications</u><br>metformin and Januvia	0	11.3-7.6
Case #28	Insulin use and other DM medication used	<u>Insulin</u><br>Levemir and Aspart<br><u>Other DM medications</u><br>metformin and glimepiride	2	9.4-8.0
Case #29	Insulin use and other DM medication used	<u>Insulin</u><br>Levemir, lispro and Aspart<br><u>Other DM medications</u><br>metformin and glipizide	3	9.8-8.0
Case #30	Insulin use and other DM medication used	<u>Insulin</u><br>glargine, and lispro,<br><u>Other DM medications</u><br>metformin, pioglitazone and glimepiride	2	12.1-7.7

Case #31	Insulin use and other DM medication used	Insulin<br>Levemir and lispro,<br>Other DM medications<br>metformin	0	9.9-7.8
Case #32	No Insulin use, but other DM medications used	Insulin<br>None<br>Other DM medications<br>Metformin, Januvia, Jardiance, glipizide	1	7.6-7.4
Case #33	No Insulin use, but other DM medications used	Insulin<br>None<br>Other DM medications<br>metformin, Steglaro, pioglitazone	2	7.6-7.0
Case #34	Insulin use and other DM medication used: Trulicity	Insulin<br>Humulin N and Novolin N<br>Other DM medications<br>Trulicity	2	12.9-7.0
Case #35	Insulin use and other DM medication used	Insulin<br>Humulin<br>Other DM medications<br>metformin and pioglitazone 15 mg	0	8.2-7.8
Case #36	No Insulin use, but other DM medications used	Insulin<br>None<br>Other DM medications<br>metformin, Jardiance, glipizide	1	7.4-6.8
Case #37	Insulin use and other DM medication used	Insulin<br>Humulin N and Novolin N<br>Other DM medications<br>Jardiance	1	10.3-7.1
Case #38	No Insulin use, but other DM medications used	Insulin<br>None<br>Other DM medications<br>pioglitazone, glyburide/metformin	0	10.3-7.7
Case #39	Insulin use and other DM medication used	Insulin<br>Aspart and Levemir<br>Other DM medications<br>metformin	0	8.1-8.0
Case #40	Insulin use and other DM medication used	Insulin<br>Aspart and Levemir<br>Other DM medications<br>metformin, Januvia, Victoza, and glipizide	4	7.3-6.9
Case #41	Insulin use and other DM medication used	Insulin<br>Levemir<br>Other DM medications<br>metformin and Januvia	0	7.5-6.8
Case #42	Insulin use and other DM medication used	Insulin<br>glargine<br>Other DM medications<br>Metformin, Jardiance, Januvia	1	16.1-8.0

Case #43	No Insulin use, but other DM medications used	<u>Insulin</u><br>None<br><u>Other DM medications</u><br>pioglitazone, glipizide, Tradjenta	3	7.3-5.2
Case #44	Insulin use only	<u>Insulin</u><br>Aspart and Levemir<br><u>Other DM medications</u><br>None	0	10.4-7.0
Case #45	Insulin use and other DM medication used	<u>Insulin</u><br>Lispro and Lantus<br><u>Other DM medications</u><br>Metformin	1	8.0-7.5
Case #46	Insulin use and other DM medication used	<u>Insulin</u><br>Lantus, Levemir, and Humulin<br><u>Other DM medications</u><br>Metformin, Januvia and glimepiride	3	11.6-7.8
Case #47	Insulin use only	<u>Insulin</u><br>glargine and lispro<br><u>Other DM medications</u><br>None	1	12.6-7.5
Case #48	Insulin use and other DM medication used	<u>Insulin</u><br>Lantus and Aspart<br><u>Other DM medications</u><br>metformin	2	8.5-7.6
Case #49	Insulin use and other DM medication used	<u>Insulin</u><br>Levemir, Humulin and Aspart<br><u>Other DM medications</u><br>metformin	2	15.6-6.8
Case #50	Insulin use and other DM medication used	<u>Insulin</u><br>Levemir<br><u>Other DM medications</u><br>metformin	1	8.7-6.2
Case #51	Insulin use and other DM medication used	<u>Insulin</u><br>Aspart and Lantus<br><u>Other DM medications</u><br>Metformin and Januvia	2	15.6-7.9
Case #52	Insulin use only	<u>Insulin</u><br>Levemir Aspart and Levemir FlexTouch<br><u>Other DM medications</u><br>None	0	7.9-7.6
Case #53	Insulin use only	<u>Insulin</u><br>Aspart and Levemir<br><u>Other DM medications</u><br>None	1	10.8-7.1
Case #54	Insulin use only	<u>Insulin</u><br>Aspart and Levemir<br><u>Other DM medications</u><br>None	2	8.3-7.1

Case #55	Insulin use and other DM medication used	<u>Insulin</u><br>glargine<br><u>Other DM medications</u><br>metformin and glipizide	0	9.7-7.2
Case #56	Insulin use and other DM medication used	<u>Insulin</u><br>Levemir, Humulin nph, Aspart<br><u>Other DM medications</u><br>metformin	3	9.2-5.9
Case #57	Insulin use and other DM medication used	<u>Insulin</u><br>Humulin<br><u>Other DM medications</u><br>metformin and glimepiride	3	15.9-6.8
Case #58	Insulin use and other DM medication used	<u>Insulin</u><br>Lispro and glargine<br><u>Other DM medications</u><br>Metformin, Trulicity, Januvia, and Tradjenta	1	9.7-8.0
Case #59	Insulin use only	<u>Insulin</u><br>Aspart and Levemir<br><u>Other DM medications</u><br>None	0	10.2-6.2
Case #60	No Insulin use, but other DM medications used	<u>Insulin</u><br>None<br><u>Other DM medications</u><br>pioglitazone, glyburide, Jardiance and metformin	3	11.2-8.0
Case #61	No Insulin use, but other DM medications used	<u>Insulin</u><br>None<br><u>Other DM medications</u><br>metformin, glipizide and Jardiance	1	9.0-7.5
Case #62	Insulin use and other DM medication used	<u>Insulin</u><br>Humulin, Aspart, and Lantus<br><u>Other DM medications</u><br>metformin and Januvia	1	9.4-6.3
Case #63	Insulin use and other DM medication used	<u>Insulin</u><br>Levemir, and Aspart<br><u>Other DM medications</u><br>glimepiride and pioglitazone	2	10.9-7.6
Case #64	Insulin use and other DM medication used	<u>Insulin</u><br>Lantus, and Basaglar<br><u>Other DM medications</u><br>glipizide, metformin and Januvia	3	9.9-7.9
Case #65	No Insulin use, but other DM medications used	<u>Insulin</u><br>None<br><u>Other DM medications</u><br>metformin and glipizide	1	8.0-7.3
Case #66	Insulin use and other DM medication used	<u>Insulin</u><br>Lantus, NovoLog, lispro, Humulin and Levemir<br><u>Other DM medications</u><br>Trulicity	4	11.0-7.5

Case #67	Insulin use and other DM medication used	Insulin<br>Levemir<br>Other DM medications<br>Trulicity, metformin and Januvia	1	9.9-7.5
Case #68	No Insulin use, but other DM medications used	Insulin<br>None<br>Other DM medications<br>metformin, glimepiride and glipizide	1	11.2-6.3
Case #69	No Insulin use, but other DM medications used	Insulin<br>None<br>Other DM medications<br>metformin and glimepiride	0	6.4-7.9
Case 70	No Insulin use, but other DM medications used	Insulin<br>None<br>Other DM medications<br>metformin, glyburide and pioglitazone	1	8.9-7.7
Case 71	Insulin use and other DM medication used	Insulin<br>Lispro, Aspart and Levemir<br>Other DM medications<br>metformin	4	5.7-5.4
Case #72	Insulin use and other DM medication used	Insulin<br>Lantus<br>Other DM medications<br>metformin	1	9.8-7.4
Case #73	Insulin use and other DM medication used	Insulin<br>Lispro, Aspart, Basaglar Kwik pen, Levemir and glargine<br>Other DM medications<br>metformin and Tradjenta	2	9.7-7.4
Case #74	Insulin use and other DM medication used	Insulin<br>Levemir and Aspart<br>Other DM medications<br>metformin, and Jardiance	1	9.7-7.4
Case #75	Insulin use and other DM medication used	Insulin<br>Levemir FlexTouch and lispro<br>Other DM medications<br>Pioglitazone, Januvia, metformin and Jardiance	4	9.2-7.6
Case #76	Insulin use and other DM medication used	Insulin<br>Levemir, glargine, and Aspart<br>Other DM medications<br>Januvia, metformin and glimepiride	5	11.0-7.6
Case #77	Insulin use and other DM medication used	Insulin<br>Humalog, Lantus, Lispro<br>Other DM medications<br>Trulicity and Metformin	3	6.7-7.3
Case #78	Insulin use and other DM medication used	Insulin<br>glargine<br>Other DM medications	1	8.4-7.1

		Metformin, glyburide, and Victoza		
Case #79	No Insulin use, but other DM medications used	Insulin<br>None<br>Other DM medications<br>metformin, glipizide, Farxiga and pioglitazone	1	8.4-6.9
Case #80	No Insulin use, but other DM medications used	Insulin<br>None<br>Other DM medications<br>metformin	0	8.6-6.1
Case #81	No Insulin use, but other DM medications used	Insulin<br>None<br>Other DM medications<br>glyburide, glyburide-metformin, metformin	2	8.4-7.9
Case #82	Insulin use and other DM medication used	Insulin<br>Levemir<br>Other DM medications<br>Metformin, Januvia, and Kombiglyze Xr 5mg-1000mg	0	6.3-7.6
Case #83	Insulin use and other DM medication used	Insulin<br>Aspart and Levemir<br>Other DM medications<br>metformin	0	10.9-8.0
Case #84	No Insulin use, but other DM medications used	Insulin<br>None<br>Other DM medications<br>metformin, glipizide, and pioglitazone	0	8.7-6.8
Case #85	No Insulin use, but other DM medications used	Insulin<br>None<br>Other DM medications<br>metformin, glipizide, and Victoza	1	7.9-7.0
Case #86	Insulin use only	Insulin<br>Lispro, Lantus and NPH<br>Other DM medications<br>None	2	9.3-7.7
Case #87	Insulin use and other DM medication used	Insulin<br>Aspart, glargine and Levemir<br>Other DM medications<br>metformin	1	13.1-7.4
Case #88	No Insulin use, but other DM medications used	Insulin<br>None<br>Other DM medications<br>glipizide-metformin, and Januvia	0	7.8-6.9
Case #89	Insulin use and other DM medication used: metformin and glipizide	Insulin<br>Basaglar, Humulin, and Levemir<br>Other DM medications<br>metformin and glipizide	4	12.3-6.3

Case #90	No Insulin use, but other DM medications used	Insulin<br>None<br>Other DM medications<br>metformin, Invokana, Jardiance/metformin and pioglitazone	1	10.3-7.6
Case #91	Insulin use and other DM medication used: metformin	Insulin<br>Lispro and Lantus,<br>Other DM medications<br>metformin	1	16.2-7.7
Case #92	Insulin use and other DM medication used	Insulin<br>glargine<br>Other DM medications<br>metformin, glimepiride and Trulicity	3	11.3-7.0
Case #93 (8034)	Insulin use only	Insulin<br>glargine<br>Other DM medications<br>None	0	8.6-7.6
Case #94	Insulin use and other DM medication used	Insulin<br>Lantus and NovoLog<br>Other DM medications<br>metformin and Januvia	2	8.8-7.2
Case #95	Insulin use and other DM medication used	Insulin<br>Aspart, Levemir and Levemir FlexTouch<br>Other DM medications<br>metformin and pioglitazone	2	8.0-7.2
Case #96	Insulin use and other DM medication used: metformin, and pioglitazone	Insulin<br>Lispro, and Levemir<br>Other DM medications<br>metformin, and pioglitazone	2	7.9-7.1
Case #97	Insulin use and other DM medication used	Insulin<br>Lispro, and Levemir<br>Other DM medications<br>Metformin and Victoza	1	11.8-7.0
Case #98	Insulin use and other DM medication used	Insulin<br>Lispro, and Levemir<br>Other DM medications<br>Metformin and glipizide	0	12.1-7.6
Case #99	Insulin use and other DM medication used	Insulin<br>Aspart and Levemir<br>Other DM medications<br>metformin	2	6.1-4.8
Case #100	No Insulin use, but other DM medications used	Insulin<br>None<br>Other DM medications<br>metformin	1	11.4-6.7
Case #101	Insulin use only	Insulin<br>glargine and NovoLog<br>Other DM medications<br>None	1	9.3-7.6

Case #102	No Insulin use, but other DM medications used	Insulin<br>None<br>Other DM medications<br>metformin, pioglitazone 30mg and Jardiance	1	12.1-7.5
Case #103	Insulin use and other DM medication used	Insulin<br>Aspart and Levemir<br>Other DM medications<br>metformin, pioglitazone and Trulicity	1	10.2-6.3
Case #104	Insulin use and other DM medication used	Insulin<br>Levemir<br>Other DM medications<br>metformin, pioglitazone, Januvia and glimepiride	1	9.2-7.7
Case #105	Insulin use and other DM medication used	Insulin<br>Aspart and Levemir FlexTouch<br>Other DM medications<br>metformin and glyburide-metformin	2	11.7-7.9
Case #106	Insulin use and other DM medication used	Insulin<br>Novolin, and Human NPH<br>Other DM medications<br>metformin and pioglitazone	2	9.3-7.8
Case #107	Insulin use and other DM medication used	Insulin<br>Aspart and Levemir<br>Other DM medications<br>metformin and pioglitazone	1	18.2-8.0
Case #108	No Insulin use, but other DM medications used	Insulin<br>None<br>Other DM medications<br>metformin and glipizide	0	8.1-7.4
Case #109	No Insulin use, but other DM medications used	Insulin<br>None<br>Other DM medications<br>metformin, Januvia, and glipizide	2	7.1-7.1
Case #110	Insulin use and other DM medication used	Insulin<br>Humalog, Aspart and Levemir<br>Other DM medications<br>metformin, Jardiance and Trulicity	4	8.5-7.5
Case #111	Insulin use and other DM medication used	Insulin<br>Levemir<br>Other DM medications<br>Metformin and pioglitazone	3	7.5-6.7
Case #112	Insulin use and other DM medication used	Insulin<br>Aspart, FlexPen and Levemir<br>Other DM medications<br>metformin, glyburide, Trulicity, and Jardiance	1	10.5-6.7
Case #113	Insulin use only	Insulin<br>Novolog, Levemir and Lantus	1	7.8-7.9

		Other DM medications<br>None		
Case #114	Insulin use and other DM medication used	Insulin<br>Aspart, and Levemir<br>Other DM medications<br>glipizide and sitagliptin	1	10.3-8.0
Case #115	Insulin use and other DM medication used	Insulin<br>Levemir<br>Other DM medications<br>metformin and glipizide	1	12.2-5.3
Case #116	Insulin use and other DM medication used	Insulin<br>Aspart and Levemir<br>Other DM medications<br>metformin	2	12.4-6.4
Case #117	No Insulin use, but other DM medications used: metformin	Insulin<br>None<br>Other DM medications<br>metformin 500mg	1	10.4-8.0
Case #118	Insulin use and other DM medication used	Insulin<br>Lispro, Aspart and Levemir<br>Other DM medications<br>metformin and glipizide	1	18.1-7.8
Case #119	No Insulin use, but other DM medications used	Insulin<br>None<br>Other DM medications<br>metformin	1	8.7-7.6
Case #120	Insulin use and other DM medication used	Insulin<br>Aspart and Levemir<br>Other DM medications<br>metformin	2	11.7-6.2
Case #121	Insulin use and other DM medication used: metformin	Insulin<br>glargine, glulisine and Human NPH<br>Other DM medications<br>metformin	4	11.1-7.4
Case #122	Insulin use and other DM medication used	Insulin<br>Humalog, and glargine<br>Other DM medication<br>metformin	1	7.6-6.8
Case #123	Insulin use and other DM medication used	Insulin<br>Levemir<br>Other DM medication<br>metformin, glimepiride, and glipizide	3	10.2-7.3
Case #124	Insulin use only	Insulin<br>Lantus and Humalog<br>Other DM medication<br>None	1	8.4-6.7
Case #125	No Insulin use, but other DM medications used	Insulin<br>None<br>Other DM medications<br>metformin and glipizide	1	7.6-6.9

Case #126	Insulin use and other DM medication used: metformin	Insulin<br>Glargine and Humulin-R<br>Other DM medication<br>metformin	0	11.8-7.8
Case #127	No Insulin use, but other DM medications used: metformin	Insulin<br>None<br>Other DM medications<br>metformin	0	9.7-6.3
Case #128	Insulin use only	Insulin<br>Levemir and lispro<br>Other DM medication<br>None	1	11.3-7.3

Table S2: Case Examples for Patients with A1c Changes to Uncontrolled Status (N=110)

Case ID	Insulin Use Pattern	Prescription Pattern Descriptions	Number of Prescriptions Discontinued	A1C Change Pre COVID to COVID era
Case #1	No Insulin use, but other DM medications used	<u>Insulin</u><br>No insulin<br><br><u>Other DM medications</u><br>glipizide, Metformin,	0	7.2-10.3
Case #2	No Insulin use, but other DM medications used	<u>Insulin</u><br>No insulin<br><br><u>Other DM medications</u><br>metformin	1	6.8-8.2
Case #3	No Insulin use, but other DM medications used	<u>Insulin</u><br>No insulin<br><br><u>Other DM medications</u><br>glipizide, metformin and empagliflozin	0	7.4-8.6
Case #4	No Insulin use, but other DM medications used	<u>Insulin</u><br>No insulin<br><br><u>Other DM medications</u><br>metformin, Trulicity, pioglitazone	0	7.7-9.5
Case #5	Insulin use and other DM medication used	<u>Insulin</u><br>Insulin Glargine<br><u>Other DM medications</u><br>glimepiride, Jardiance, Metformin, Trulicity and Januvia	3	6.3-11.7
Case #6	Insulin use and other DM medication used:	<u>Insulin</u><br>Aspart, Novolin, NPH, and Levemir<br><u>Other DM medications</u><br>Tradjenta, pioglitazone, metformin	4	9.4-8.2
Case #7	Insulin use only	<u>Insulin</u><br>Levemir<br><u>Other DM medications</u><br>None	0	8.8-12.1
Case #8	No Insulin use, but other DM medications used	<u>Insulin</u><br>None<br><br><u>Other DM medications</u><br>metformin 1000 mg and Januvia 100 mg	0	7.9-9.5

Case #9	Insulin use and other DM medication used	Insulin<br>Lantus<br>Other DM medications<br>metformin and Glimepiride	0	11.6-8.4
Case #10	Insulin use and other DM medication used	Insulin<br>Aspart, NPH, and Humulin<br>Other DM medications<br>metformin	0	10.2-8.1
Case #11	No Insulin use, but other DM medications used	Insulin<br>None<br>Other DM medications<br>metformin		6.8-9.5
Case #12	Insulin use, but other DM medications used:	Insulin<br>Levemir<br>Other DM medications<br>metformin	2	6.1-9.2
Case #13	Insulin use and other DM medication used	Insulin<br>Levemir and Aspart,<br><br>Other DM medications<br>metformin	0	9.5-8.2
Case #14	Insulin use and other DM medication used	Insulin<br>glargine<br>Other DM medications<br>metformin 1	0	10.2-12.0
Case #15	No Insulin use, but other DM medications used	Insulin<br>None<br>Other DM medications<br>Jardiance and metformin	1	7.6-10.2
Case #16	Insulin use and other DM medication used	Insulin<br>Levemir and Aspart<br>Other DM medications<br>Victoza and pioglitazone	1	12.6-12.1
Case #17	Insulin use and other DM medication used	Insulin<br>Aspart and Lantus<br>Other DM medications<br>metformin	0	8.5-8.5
Case #18	Insulin use and other DM medication used	Insulin<br>Levemir<br>Other DM medications<br>metformin and Januvia	0	9.6-9.2
Case #19	Insulin use only	Insulin<br>Lantus<br>Other DM medications<br>None	0	8.4-9.5
Case #20	Insulin use and other DM	Insulin<br>Novolog, Aspart and Levemir<br>Other DM medications	3	14.2-8.2

	medication used	metformin		
Case #21 (	No Insulin use, but other DM medications used	Insulin<br>None<br>Other DM medications<br>metformin, glipizide, and Pioglitazone	0	9.1-10
Case #22	No Insulin use, but other DM medications used	Insulin<br>None<br>Other DM medications<br>metformin and glipizide	0	7.5-10.2
Case #23	Insulin use only	Insulin<br>glargine<br>Regular<br>Other DM medications<br>None	0	6.1-8.1
Case #24	No Insulin use, but other DM medications used	Insulin<br>None<br>Other DM medications<br>metformin and pioglitazone	2	8.3-8.3
Case #25	No Insulin use, but other DM medications used	Insulin<br>None<br>Other DM medications<br>metformin and glyburide	0	7.0-9.9
Case #26	No Insulin use, but other DM medications used	Insulin<br>None<br>Other DM medications<br>metformin and glipizide	1	6.3-9.3
Case #27	No Insulin use, but other DM medications used	Insulin<br>None<br>Other DM medications<br>metformin	1	9.5-8.6
Case #28	No Insulin use, but other DM medications used	Insulin<br>None<br>Other DM medications<br>metformin, glimepiride, Januvia and Janumet	3	8.3-8.3
Case #29	Insulin use and other DM medication used: Metformin	Insulin<br>Levemir and Lispro<br>Regular<br>Other DM medications<br>Metformin 1000 mg	1	9.0-11.0
Case #30	Insulin use and other DM	Insulin<br>Levemir and Aspart	2	10.2-9.1

	medication used	Regular<br>Other DM medications<br>metformin		
Case #31	No Insulin use, but other DM medications used:	Insulin<br>None<br>Other DM medications<br>metformin	0	7.7-8.7
Case #32	Insulin use and other DM medication used	Insulin<br>Levemir<br>Other DM medications<br>metformin, pioglitazone, glipizide	3	11.9-9.6
Case #33	Insulin use and other DM medication used	Insulin<br>glargine<br>Other DM medications<br>metformin, Trulicity, and glipizide	2	7.3-10.3
Case #34	Insulin use and other DM medication used	Insulin<br>Aspart and Levemir<br>Other DM medications<br>metformin	0	9.1-9.1
Case #35	Insulin use and other DM medication used	Insulin<br>Aspart and Levemir<br>Other DM medications<br>Trulicity	0	10.4-12.1
Case #36	Insulin use and other DM medication used	Insulin<br>Levemir<br>Other DM medications<br>metformin and glipizide	2	7.3-9.7
Case #37	No Insulin use, but other DM medications used	Insulin<br>None<br>Other DM medications<br>metformin, Jardiance, and glyburide	1	10.5-8.2
Case #38	Insulin use and other DM medication used	Insulin<br>Aspart and Levemir<br>Other DM medications<br>glimepiride	2	10.7-8.4
Case #39	No Insulin use, but other DM medications used	Insulin<br>None<br>Other DM medications<br>metformin, Januvia, and Trulicity	0	9.6-10.7
Case #40	Insulin use and other DM medication used	Insulin<br>Humulin<br>Other DM medications<br>glimepiride and metformin	1	9.6-8.2
Case #41	Insulin use and other DM	Insulin<br>Levemir	1	6.9-8.2

	medication used	Other DM medications<br>glipizide and metformin		
Case #42	Insulin use and other DM medication used	Insulin<br>Levemir<br>Other DM medications<br>metformin	0	8.3-8.7
Case #43	No Insulin use, but other DM medications used	Insulin<br>None<br>Other DM medications<br>metformin, pioglitazone, Januvia, and glipizide	2	10.3-8.3
Case #44	Insulin use and other DM medication used	Insulin<br>Levemir<br>Other DM medications<br>metformin and Trulicity	2	7.2-14.0
Case #45	Insulin use only<br>Lancets	Insulin<br>Humalog and Levemir Regular<br>Other DM medications<br>None	2	11.6-9.7
Case #46	Insulin use and other DM medication used	Insulin<br>Lantus<br>Other DM medications<br>metformin	0	9.0-9.4
Case #47	Insulin use and other DM medication used	Insulin<br>Novolog and Levemir<br>Other DM medications<br>metformin	0	6.0-11
Case #48	No Insulin use, but other DM medications used	Insulin<br>None<br>Other DM medications<br>metformin	1	8.0-11.9
Case #49	Insulin use and other DM medication used	Insulin<br>Levemir<br>Other DM medications<br>metformin	1	6.5-8.8
Case #50	No Insulin use, but other DM medications used	Insulin<br>None<br>Other DM medications<br>metformin, Invokana, and glipizide	1	7.5-8.5
Case #51	No Insulin use, but other DM medications used	Insulin<br>None<br>Other DM medications<br>metformin	0	7.2-11.7
Case #52	Insulin use and other DM medication used	Insulin<br>Aspart and glargine<br>Other DM medications<br>metformin and glyburide	1	13.5-13.2
Case #53	Insulin use only	Insulin<br>NPH, Aspart, and Levemir<br>Other DM medications<br>None	2	7.3-8.6

Case #54	No Insulin use, but other DM medications used	Insulin<br>None<br>Other DM medications<br>metformin, glipizide, and pioglitazone	1	8.3-8.2
Case #55	No Insulin use, but other DM medications used	Insulin<br>None<br>Other DM medications<br>metformin and glimepiride	0	8.9-14.1
Case #56	Insulin use and other DM medication used	Insulin<br>Levemir<br>Other DM medications<br>metformin and Trulicity	0	8.6-10.6
Case #57	No Insulin use, but other DM medications used	Insulin<br>None<br>Other DM medications<br>metformin, Januvia, and glimepiride	2	8.9-9.8
Case #58	Insulin use and other DM medication used	Insulin<br>Levemir FlexTouch, Levemir<br>Other DM medications<br>Metformin and pioglitazone	2	7.8-9.1
Case #59	No Insulin use, but other DM medications used	Insulin<br>None<br>Other DM medications<br>metformin and glipizide	0	8.3-9.7
Case #60	Insulin use and other DM medication used	Insulin<br>Lantus and lispro<br>Other DM medications<br>metformin	3	8.0-8.2
Case #61	Insulin use and other DM medication used	Insulin<br>Levemir and Aspart<br>Other DM medications<br>glipizide-metformin	1	8.9-11.6
Case #62	Insulin use and other DM medication used	Insulin<br>Levemir and Aspart<br>Other DM medications<br>glipizide, Trulicity, metformin and pioglitazone	5	16.2-14.1
Case #63	Insulin use and other DM medication used	Insulin<br>Levemir<br>Other DM medications<br>metformin and pioglitazone	2	10.1-8.7
Case #64	Insulin use and other DM medication used	Insulin<br>Levemir FlexTouch<br>Other DM medications<br>metformin	0	7.1-10.5
Case #65	No Insulin use, but other DM medications used	Insulin<br>None<br>Other DM medications<br>metformin	1	5.7-8.8
Case #66	No Insulin use, but other DM	Insulin<br>None	2	7.3-8.6

	medications used	<u>Other DM medications</u><br>Jardiance, Janumet, glyburide, Steglatro, and glyburide-metformin		
Case #67	No Insulin use, but other DM medications used	<u>Insulin</u><br>None<br><u>Other DM medications</u><br>metformin, glyburide, and pioglitazone	0	7.3-8.3
Case #68	No Insulin use, but other DM medications used: metformin, pioglitazone and glimepiride	<u>Insulin</u><br>None<br><u>Other DM medications</u><br>metformin, pioglitazone and glimepiride	0	11.8-8.3
Case #69	Insulin use and other DM medication used: metformin	<u>Insulin</u><br>glargine, Aspart and Lispro<br><u>Other DM medications</u><br>metformin	1	7.4-9.2
Case #70	No Insulin use, but other DM medications used	<u>Insulin</u><br>None<br><u>Other DM medications</u><br>metformin, Trulicity and Januvia	1	6.3-9.3
Case #71	No Insulin use, but other DM medications used	<u>Insulin</u><br>None<br><u>Other DM medications</u><br>metformin, glyburide, Trulicity and pioglitazone	1	10.9-9.6
Case #72	No Insulin use, but other DM medications used	<u>Insulin</u><br>None<br><u>Other DM medications</u><br>metformin, and pioglitazone	2	7.5-8.1
Case #73	No Insulin use, but other DM medications used	<u>Insulin</u><br>None<br><u>Other DM medications</u><br>metformin, glipizide, and pioglitazone	1	8.2-9.1
Case #74	Insulin use and other DM medication used	<u>Insulin</u><br>Aspart and Levemir<br><u>Other DM medications</u><br>metformin and Jardiance	0	7.8-8.7
Case #75	Insulin use and other DM medication used	<u>Insulin</u><br>Levemir<br><u>Other DM medications</u><br>metformin	2	7.7-9.2
Case #76	Insulin use only	<u>Insulin</u><br>Lispro and Lantus<br><u>Other DM medications</u><br>None	1	14.1-9.8

Case #77	Insulin use and other DM medication used	Insulin<br>Levemir<br>Other DM medications<br>metformin and glipizide	1	10.3-8.9
Case #78	Insulin use and other DM medication used	Insulin<br>glargine and Lispro<br>Other DM medications<br>metformin and pioglitazone	0	9.6-8.2
Case #79	Insulin use and other DM medication used	Insulin<br>Humulin 70/30, Humulin NPH<br>Other DM medications<br>metformin	1	8.4-8.3
Case #80	No Insulin use, but other DM medications used	Insulin<br>None<br>Other DM medications<br>metformin and Jardiance	0	7.0-11.6
Case #81	Insulin use and other DM medication used	Insulin<br>Humalog Kwikpen<br>Other DM medications<br>metformin	0	6.7-8.5
Case #82	Insulin use and other DM medication used	Insulin<br>glargine<br>Other DM medications<br>pioglitazone	0	7.0-8.5
Case #83	No Insulin use, but other DM medications used	Insulin<br>None<br>Other DM medications<br>metformin	0	10.5-10.0
Case #84	No Insulin use, but other DM medications used	Insulin<br>None<br>Other DM medications<br>metformin and glipizide	0	8.1-9.2
Case #85	No Insulin use, but other DM medications used	Insulin<br>None<br>Other DM medications<br>metformin, Januvia, and glyburide	1	8.0-8.4
Case #86	Insulin use and other DM medication used: metformin	Insulin<br>Aspart and Levemir<br>Other DM medications<br>metformin	0	11.7-10.8
Case #87	No Insulin use, but other DM medications used: metformin and glipizide	Insulin<br>None<br>Other DM medications<br>metformin and glipizide	1	7.2-9.4
Case #88	No Insulin use, but other DM medications used:	Insulin<br>None<br>Other DM medications<br>metformin and glipizide	1	6.6-10.1

	metformin and glipizide			
Case #89	No Insulin use, but other DM medications used: metformin	Insulin<br>None<br>Other DM medications<br>metformin	0	6.9-9.4
Case #90	No Insulin use, but other DM medications used	Insulin<br>None<br>Other DM medications<br>glyburide-metformin	0	6.5-8.1
Case #91	No Insulin use, but other DM medications used	insulin<br>None<br>Other DM medications<br>glipizide and metformin	1	9.8-10.8
Case #92	No Insulin use, but other DM medications used	insulin<br>None<br>Other DM medications<br>metformin	0	7.9-9.9
Case #93	Insulin use and other DM medication used: metformin and glimepiride	Insulin<br>glargine<br>Other DM medications<br>metformin and glimepiride	0	9.9-9.9
Case #94	Insulin use and other DM medication used:	Insulin<br>Levemir<br>Other DM medications<br>metformin, pioglitazone and Onglyza	0	8.3-9.9
Case #95	Insulin use and other DM medication used	Insulin<br>Aspart and NovoLog flexpen, Levemir<br>Other DM medications<br>metformin	0	9.3-8.1
Case #96	No Insulin use, but other DM medications used	insulin<br>None<br>Other DM medications<br>metformin and glipizide	0	10.6-8.2
Case #97	No Insulin use, but other DM medications used	insulin<br>None<br>Other DM medications<br>metformin and Januvia	1	6.5-8.4
Case #98	Insulin use and other DM medication used	Insulin<br>Lantus and Basaglar<br>Other DM medications<br>metformin and Januvia	2	12.3-8.7
Case #99	No Insulin use, but other DM medications used	insulin<br>None<br>Other DM medications<br>metformin, pioglitazone and glipizide	1	9.0-9.2

Case #100	No Insulin use, but other DM medications used	insulin<br>None<br>Other DM medications<br>Metformin and Jardiance	0	9.8-9.5
Case #101	Insulin use only	Insulin<br>Humulin<br>Other DM medications<br>None	1	12.4-12.0
Case #102	No Insulin use, but other DM medications used	Insulin<br>None<br>Other DM medications<br>metformin, and glipizide	1	7.9-13.3
Case #103	Insulin use and other DM medication used	Insulin<br>glargine<br>Other DM medications<br>metformin and Jardiance	0	7.4-10.5
Case #104	No Insulin use, but other DM medications used	Insulin<br>None<br>Other DM medications<br>metformin and glipizide	0	11.3-8.2
Case #105	Insulin use and other DM medication used,	Insulin<br>Humulin-R<br>Other DM medications<br>metformin, glyburide and pioglitazone	1	11.3-9.3
Case #106	Insulin use and other DM medication used	Insulin<br>Levemir<br>Other DM medications<br>metformin and Jardiance	0	10.1-8.4
Case #107	No Insulin use, but other DM medications used	Insulin<br>None<br>Other DM medications<br>metformin	0	6.4-8.1
Case #108	Insulin use and other DM medication used	Insulin<br>Levemir<br>Other DM medications<br>metformin and glimepiride	1	12.0-8.2
Case #109	Insulin use and other DM medication used	Insulin<br>Lantus<br>Other DM medications<br>metformin	1	10.9-9.3
Case #110	Insulin use and other DM medication used	Insulin<br>Aspart, NPH, Humulin-R and Levemir<br>Other DM medications<br>metformin	5	8.4-11.1

* Cases with A1c changes were defined as controlled (improved ≥8% to <8%) or uncontrolled (worsened ≤8% to > 8%)
